# Evaluation of the ideal length of the Seldinger needle for internal jugular vein catheter placement

**DOI:** 10.1038/s41598-022-06287-4

**Published:** 2022-02-17

**Authors:** Clara M. Wenzel, Torsten M. Meyer, Dietrich Stoevesandt, Heike Kielstein, Jan T. Kielstein

**Affiliations:** 1 Medical Clinic V, Nephrology | Rheumatology | Blood Purification, Academic Teaching Hospital Braunschweig, Salzdahlumer Straße 90, 38126 Braunschweig, Germany; 2 Institute of Anatomy and Cell Biology, Medical Faculty, Martin Luther University Halle-Wittenberg, Halle (Saale), Germany; 3 Department and Outpatient Clinic for Radiology, University Hospital Halle (Saale), Halle (Saale), Germany

**Keywords:** Anatomy, Disease prevention

## Abstract

Placement of central venous catheters (CVC) into the internal jugular vein represents a routine clinical intervention. The periprocedural complication rate ranges from 5 to 20% and can be reduced by ultrasound guidance, training of residents and other measures. We aimed to proof that the average Seldinger needle is too long, increasing the risk of periprocedural injury, best epitomized in the stellate ganglion injury/irritation. The *first part* of the study was an online market analysis to investigate the standard needle length currently offered as part of the CVC placement sets. The *second part* of the study involved 35 hospitalized patients (14 female; median age 74.5 years). In those the distance between the skin and the internal jugular vein as well as the diameter of the internal jugular vein was measured by ultrasound in both, supine position as well as 45° semi-sitting position. In the *third part* of the study 80 body donors (45 female; median age 83.0 years) preserved by the ethanol/formaldehyde method were studied. In those the distance and angle between the typical landmark for insertion of the Seldinger needle for internal jugular vein catheter placement to the stellate ganglion was measured. The median [interquartile range] Seldinger needle length was 7 [4.0–10.0] cm. In the examined patients the maximum distance between the skin and the internal jugular vein was 1.87 cm. The minimum distance was 0.46 cm and the median distance averaging supine and 45° position was 1.14 [0.94–1.31] cm. Regarding the body donors the median distance from the insertion point of the internal jugular vein to the stellate ganglion was longer in men 5.5 [4.95–6.35] cm than in women 5.2 [4.7–5.9] (p = 0.031 unpaired t-test). With 7 cm average length the Seldinger needle currently sold as part of CVC sets is long enough to physically reach the stellate ganglion, not to mention more proximal structures. A shorter needle length would be sufficient to reach the internal jugular vein even in obese patients and with a small insertion angle while minimizing the possibility to cause severe injury as structures like the pleura and the stellate ganglion could not be reached by shorter needles.

## Introduction

Although first mentioned in 1929^1^ placement of central venous catheters (CVCs) developed into a routine clinical intervention after publication of an ingenious technique first described by Dr. Sven Ivar Seldinger in 1953^2^. Market research estimates that 27 million CVCs have been placed globally in 2020^3^. Given this impressive number even a low insertion complication rate of 5–20% including hematoma, venous perforation, arterial puncture and pneumothorax adds up to a high case injury burden^4,5^. If cardiac arrhythmias are added as non-structural peri-procedural side effects, the complication rate can approach almost 50%^6^. Although a larger study found no differences between anatomic sites for either total mechanical or total delayed complications^7^, site specific variations do occur. Pneumothorax and hemothorax are more frequent in subclavian vein catheterization as compared to internal jugular vein which is however more prone to be associated with arterial puncture^8^. This holds especially true for the rare Horner syndrome caused by internal jugular vein catheterization^9^ that accounts for up to 5% of the complications^10^. A meta-analysis of seventeen prospective comparative trials with data on 2085 jugular and 2428 subclavian catheters revealed a complication rate of Horner syndrome of 5%^11^. Analysing 22 case reports showed the following risk factors for occurrence of the Horner syndrome: right internal jugular vein puncture, repeated attempts of puncture, use of anatomic landmark technique, accidental carotid artery puncture and hematoma formation^9^. Many strategies had been undertaken to reduce the periprocedural complication rate. A high level of experience is associated with less iatrogenic injuries^12^. Limiting insertion attempts to a maximum of three seems also to be a prudent approach as the incidence of mechanical complications after more than three tries is six times higher than after one attempt^13^.

Two-dimensional ultrasound has been shown to be superior in safety and quality compared to anatomical landmark techniques^14^. During internal jugular vein catheterization, ultrasound guidance reduces the number of mechanical complications as well as the procedure time^15,16^. Also, simulation of the procedure improves performance, which holds true for manikin training under the guidance and feedback of an experienced observer, as well as quantitative feedback from the personalized dynamic haptic robotic trainers learning interface^17^.

One variable that has not been addressed so far in terms of CVC placement complication is the needle length. Currently most of the CVC sets include long insertion needles to guarantee access to the three most commonly anatomic insertion sites: internal jugular vein, subclavian vein and femoral vein. New technologies using three-dimensional tracking of the needle tip in a phantom showed that the path length of the needle tip from the needle's entry point into the skin to its end point inside the vessel was almost 3 times as long in untrained residents as compared to trained residents. This is mainly due to frequent and ample correction movements by the untrained in the “tissue”^17^. Hence with a longer needle there is a potentially higher risk if this needle is inserted deeply or repositioned frequently.

The stellate ganglion, which can be injured during central venous line placement is also known as the cervicothoracic ganglion. It is a large structure of up to 10 by 20 mm, formed by the fusion of the inferior cervical ganglion and the first thoracic ganglion. It is positioned at the level of C7, anterior to the transverse process of C7 and the neck of the first rib, superior to the cervical pleura and just below the subclavian artery. Injury of the ganglion disrupting the sympathetic nerve supply results in a syndrome first described and later named after the Swiss ophthalmologist Johann Friedrich Horner in 1869^18^. Horner syndrome consisting of ptosis, miosis, and facial anhidrosis and an enophthalmos on the same side as the lesion of the sympathetic trunk.

The underlying idea of our study is that optimal, i.e. reduced needle length, would minimize the unintentional puncture of vital structures and reduction of periinterventional morbidity. This we aimed to epitomize by using a 7.0 cm long Seldinger needle to intentionally puncture the stellate ganglion in a large number of body donors.

## Methods

*First part* of the study was an online market analysis to investigate the standard needle length currently offered as part of the CVC placement sets. A google search with the search terms “central venous catheter”; “Seldinger needle”; “puncture needle” was used to identify companies that are active in this regard in Germany. Eleven companies that had been identified in the online search to produce Seldinger needles were contacted.

The *second part* of the study involved 35 hospitalized patients of our clinic (14 female) with a median age of 74.5 years. During routine ultrasound examinations the distance from the skin to the internal jugular vein and the diameter of the internal jugular vein at level of the thyroid cartilage were measured by ultrasound (Toshiba Aplio 500 PVT-712BT using a linear ultrasound transducer probe) in both, supine position as well as 45° semi-sitting position and on both sides. Patient characteristics like body mass index (BMI) and age were also recorded. To evaluate a minimum required length for the Seldinger needle to safely reach the internal jugular vein based on those ultrasound parameters an insertion angle of 45° as well as half of the vein’s diameter were included into the calculation.

**Figure 1:**
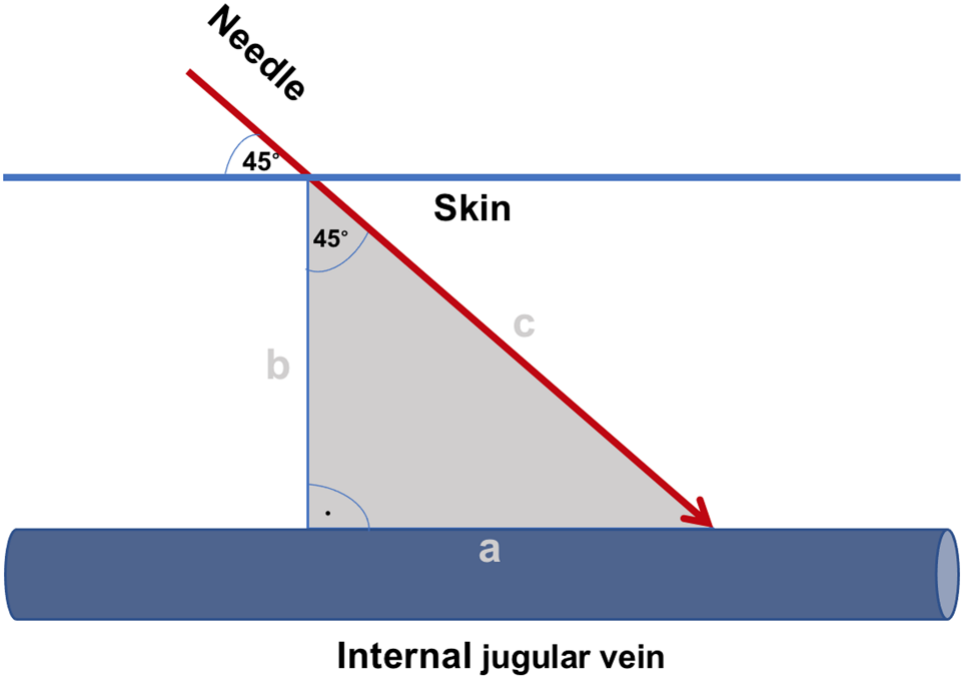
To calculate the minimum required Seldinger needle length to safely reach the middle of the lumen of the internal jugular vein, the ultrasound skin–vein distance and an insertion angel of the needle of 45° were used for the following calculation. Assuming an isosceles triangle the distance from skin to the internal jugular vein (b) at a 90° angle was measured by ultrasound. To calculate the distance from the skin to the internal jugular vein at a 45° angle (c) Pythagoras’ theorem (a^2^ + b^2^ = c^2^) was applied.

To take the puncture angle of 45° into account an isosceles triangle was assumed with distance a and b of which distance b corresponded to the measured distance from skin to the internal jugular vein at an 90° angle done by ultrasound. To include a puncture angle of 45° and therefore calculate distance c, Pythagoras’ theorem (a^2^ + b^2^ = c^2^) was applied (Fig. 1).

In the *third part* of the study 80 embalmed body donors (45 F; median age 83.0 years, range 56 to 97 years) in the Institute of Anatomy and Cell Biology in Halle (Saale) were studied. Written informed consent for scientific investigations in general is given by all body donors prior to death at the Institute of Anatomy and Cell Biology. The cadavers investigated were preserved as described previously^19^, using a solution containing ethanol (77%), unbuffered formalin (3%), glycerine, and distilled water (~ 9%, respectively). The procedure includes intravascular embalming for 6–8 h, embalming in a solution bath for 6–8 weeks, and storage at 2–4 °C for up to two years. Body donors of the annual dissection course were examined for four years. Due to removal of the lungs and the chest wall the stellate ganglion, a sympathetic ganglion formed by the fusion of the inferior cervical ganglion and the first thoracic (superior thoracic sympathetic) ganglion was visible at the level of C7, anterior to the transverse process of C7 and the neck of the first rib, superior to the cervical pleura and just below the subclavian artery. The internal jugular vein was not covered by skin or subcutaneous tissue. The insertion point of the Seldinger needle was chosen as described previously^8^ at the apex of the triangle formed by the heads of the sternocleidomastoid muscle and the clavicle. From the insertion point the needle was advanced until the stellate ganglion was penetrated (visible from caudal). The distance from the needle tip to the tissue surface was measured by deducting the visible needle length from 7.0 cm, i.e. the total length of the Seldinger needle.

### Statistics

GraphPad Prism 9 (San Diego, CA, USA) was used for statistical analysis. Data are reported as median and range unless otherwise stated. For all tests used a significance level of p < 0.05 was considered significant.

### Ethics

Written informed consent for scientific investigations in general is given by all body donors prior to death at the Institute of Anatomy and Cell Biology. This is approved by the Ethics Committee of the Martin Luther University Halle Wittenberg, Germany. The study in patients was approved by the internal review Board of the Ärztekammer Niedersachsen (Bo/47//2021). Patients gave their written informed consent to the processing of the data according to the General Data Protection Regulation of the European Union.

## Results

**Figure 2:**
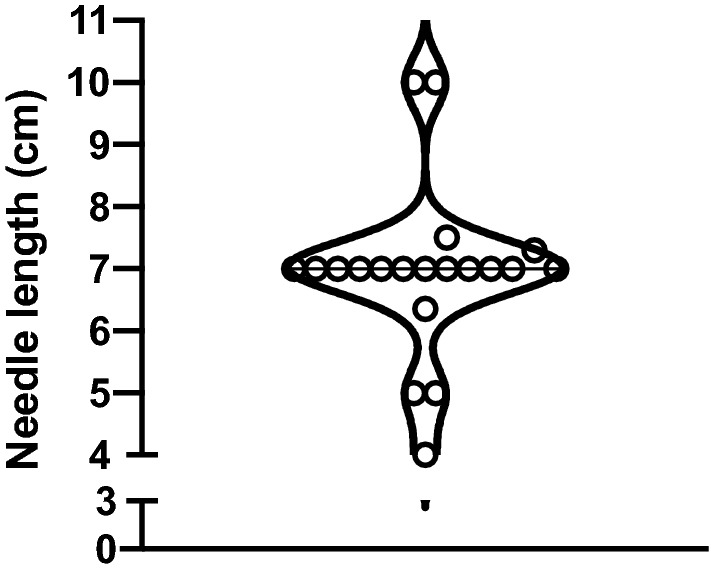
Violin plot of the needle length of Seldinger needles in the market research (n = 20).

In the *first part* of the study, our market analysis, 20 different needles from eleven different companies were analysed. The median [IQR] needle length was 7.0 [4.0–10.0] cm (Fig. 2).

During the *second part* of the study, sonographic examinations of the internal jugular veins on both sides in 35 patients (14 female) were performed. The median age was 74.5 years with a range from 28 to 89 years. The median [IQR] skin-to-vein distance of both, right and left IJV was wider in 45° position (1.17 [0.98–1.34] cm) as compared to the supine position (1.13 [0.95–1.31] cm), p = 0.0459 in the paired t-test. The median [IQR] distance from the skin to the right and left IJV was 1.15 [0.92–1.33] and 1.08 [0.93–1.34] cm respectively, which was not significantly different in the paired t-test.

**Figure 3:**
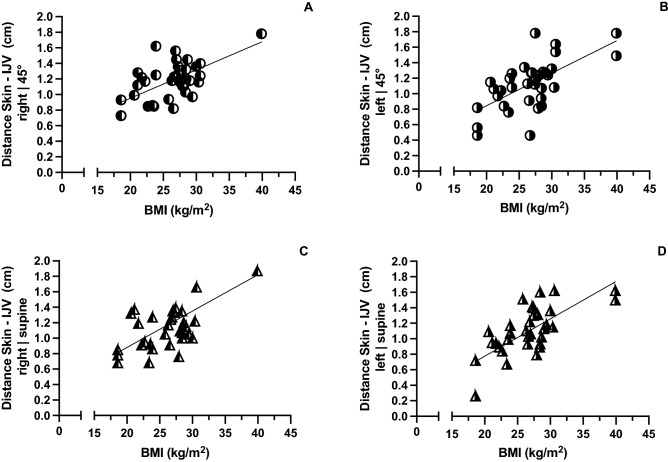
(**A**–**D**) Distance between skin and the internal jugular vein correlated with the BMI at the right and left side with an 45° angle of the upper body (Pearson r of 0.66 (**A**) and 0.638 (**B**) respectively; p < 0.0001) as well as on the right and left side in supine position (Pearson r of 0.6369 (**C**) and 0.71 (**D**) respectively; p < 0.0001).

**Figure 4:**
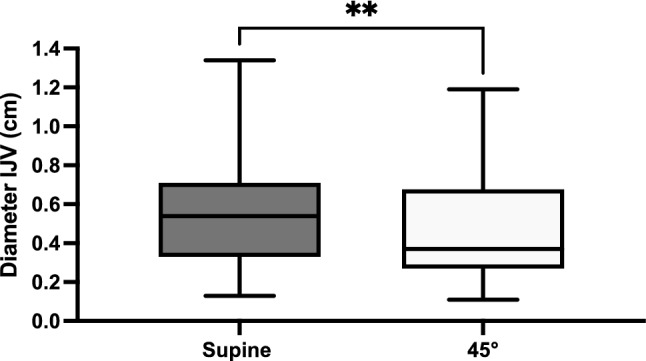
The median [IQR] diameter of the internal jugular vein was wider in the supine position (0.58 [0.37–0.72] mm) as compared to the 45° upright position (0.35 [0.26–0.63] mm) (**p = 0.0076, paired t-test).

The correlation of the distance between skin and the IJV and the patient's BMI was maintained regardless of the position of the upper body or the side of the neck **(**Fig. 3A–D). The median [IQR] diameter of the IJV in the supine position was significantly wider 0.58 [0.37–0.72] cm than in the 45° upright position 0.35 [0.26–0.63] (Fig. 4).

The insertion angle of the needle has an impact on the distance the needle tip has to travel underneath the skin to reach the IJV. A 90° angle would allow the shortest distance from the skin to the IJV and an angel of > 0° would require a longer distance. To account for this, data collected during the sonographic examinations, which represent a 90° angle, were used to calculate the distance of the needle tip that would be necessary at an insertion angle of 45° as follows:

$${\text{a}}^{{2}} + {\text{ b}}^{{2}} = {\text{ c}}^{{2}}$$

$${1}.{15}^{{2}} + { 1}.{15}^{{2}} = { 2}.{645}$$

$${\text{c }} = \sqrt {2.645}$$

$${\text{c }} = { 1}.{63}\;{\text{cm}}$$

To this value for each skin-vein-distance ½ of the vein’s diameter was added so that an inserted needle would have been inserted safely into the vein’s center.

**Figure 5:**
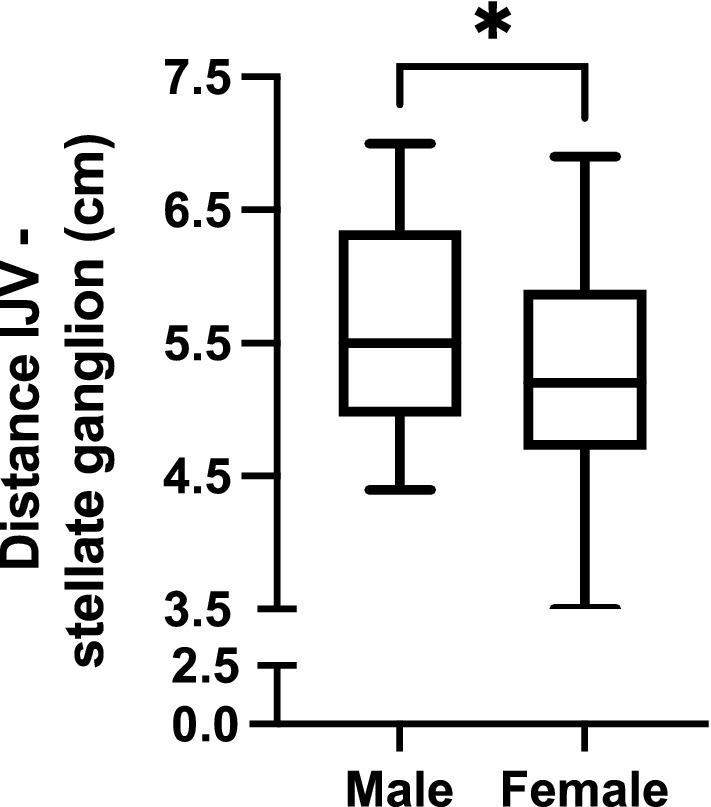
The median [IQR] distance from the IJV to the stellate ganglion was longer in male body donors (5.5 [4.95–6.35] cm) than in female body donors (5.2 [4.7–5.9] cm; *p = 0.0304, unpaired t-test).

For the *third part* of the study including 80 body donors, the median [IQR] distance from the IJV to the stellate ganglion was longer in male 5.5 [4.95–6.35] cm than in female 5.2 [4.7–5.9] cm body donors (p = 0.0304, unpaired t-test) (Fig. 5). Analysing all body donors together the median [IQR] right IJV to the stellate ganglion was 5.4 [4.9–6.0] cm. There was neither a relationship between the distance from vein to stellate ganglion to the body length nor to the age of the body donors.

## Discussion

Most commercially available central venous catheter sets are supplied with a conventional 7.0 cm Seldinger needle. As the distance from the skin to the middle of the IJV using a 45° angle was < 2 cm in 89% of our patients, there is 5 cm "unnecessary" needle length of potential harm. This was supported by the fact that a 7 cm needle was long enough to reach the Stellate ganglion in all embalmed body donors. Furthermore, the distance from the skin to the IJV depends on the BMI, a variable that is universally available and might help the clinician that has no access to sonography for guidance.

### Needle length

The median Seldinger needle length in commercial central venous catheterkits is 7 cm. While this length might be required to reach the femoral vein of an obese adult it is certainly not necessary to reach the IJV. The maximum skin-to-vein distance in our patients was 1.87 cm, the minimum distance was 0.46 cm. One limitation is the fact that we only examined the required needle length to injure the right stellate ganglion, although side-dependent differences in the topography can occur, as shown for the vagal nerve^20^.

Several approaches had been taken to avoid injury of an unnecessary long Seldinger needle. Creating indenting markings on the existing unmarked introducer needle has been shown to improve IJV cannulation and decrease the complications^21^. In a prospective randomized study it could be shown that a guard which can be slid and fixed over the needle at a desired length thus limiting maximum insertion length, improved successful cannulation in the first attempt and decreased posterior venous wall puncture as well as common carotid artery puncture^22^.

### BMI and depth of the IJV

We are not aware of a study that examined the depth of the IJV and the BMI in adults. An older study examined the relationship of the depth of the internal jugular vein (IJV) to weight in children and found an r^2^ of 0.379 ^23^.

### Position of the upper body and diameter of the IJV

Increasing the size of the internal jugular vein by positioning of the upper body has been shown previously^24^ and could be confirmed by our study. Hence proper positioning of the upper body helps to widen the IJV facilitating the puncture of the vessel. This becomes especially important if patients are unable to perform the Valsalva manoeuvre which also leads to distension of the IJV. So whenever supine positioning is possible it should be preferred over 45° upright position of the upper body.

### Length does matter: a plea for a shorter Seldinger needle

The average skin-to-vein distance of both, right and left IJV, in 45° and 0° position was 1.14 cm and correlated with the patient's BMI. The median diameter of the vessel was 0.48 cm and changed depending on the position of the patient and was significantly larger in the supine position with a median of 0.58 cm than in the 45° position where the median was 0.35 cm. Even with a small insertion angle of 23° a 4 cm needle would have been sufficient to safely reach the middle of the IJV in 92% of all our patients.

The potential harm of an average Seldinger needle length of 7 cm was epitomized by the fact that the median distance from the vein to the stellate ganglion in embalmed body donors was 5.4 cm. Taking into account that the skin and subcutaneous tissue of the body donors were already dissected the distance between skin and vein from the ultrasound study in patients has to be added to that distance. Assuming that the median of the distance between skin and vein is added at least in more than half (54%) of the body donors the Seldinger needle would have been long enough to hit the stellate ganglion. If one assumes that there could be an injury by a small hematoma close to the stellate ganglion a Horner syndrome would be possible in even more patients. This should not come to anybody’s surprise as for the Stellate ganglion block a 9 cm needle is recommended^25^. Given that fact that a large point prevalence study showed that the internal jugular vein was the preferred vessel for central venous lines, used in 79% of the patients, followed by subclavian vein (10.6%) and the femoral vein (6.4%)^26^ substituting the 7.0 cm Seldinger needle by a 5.0 cm needle would be a prudent approach.

In conclusion we postulate that a shorter Seldinger needle for placing a CVC into the IJV would be a prudent approach that would allow safe positioning of the guide wire while reducing the risk of procedure related injuries, as the injury of the stellate ganglion. In addition, shortening the Seldinger needle by 2 cm or more could safe an enormous amount of medical grade steel, thus conserving limited resources. Using 1.4301/X5CrNi18-10 steel, shorting 20 million Seldinger needles by 2 cm would save about 2100 kg steel per year.
